# The impact of age on a mitoxantrone-based tumor vaccine

**DOI:** 10.1186/2051-1426-1-S1-P234

**Published:** 2013-11-07

**Authors:** Keven Stonewall, Cecilia Quintero, Andrew Zloza, Howard Kaufman, Carl Ruby

**Affiliations:** 1 Department of Surgery, Rush University Medical Center, Chicago, IL, USA; 2 Department of Internal Medicine, Rush University Medical Center, Chicago, IL, USA; 3 Department of Immunology/Microbiology, Rush University Medical Center, Chicago, IL, USA

## 

Several chemotherapies, including the anthracycline analog mitoxantrone, are known to induce an immunogenic cell death in tumor cells that can produce a protective anti-tumor immune response in mice. Although the anti-tumor immune response mediated by mitoxantrone tumor cell death has been well described, it is not fully understood what effect age has on these responses. Our objectives were to 1) determine if older mice (12 month old) produced a protective immune response following immunization with mitoxantrone-treated CT26 colon carcinoma cells and 2) determine the age-related cellular and molecular deficiencies that could affect the generation of the protective immune response. CT26 cells treated in vitro with mitoxantrone (1.0 μM) and injected s.c. into an immune competent two-month old mouse resulted in significant protection from a secondary tumor challenge with untreated CT26 (~75% survival). However, older mice failed to generate a protective immune response and succumbed to the secondary tumor challenge (0% survival). To determine the age-related cellular and molecular dysfunction, vaccine-draining lymph nodes (vdLNs) and the vaccination site were harvested and immune cells assessed. There was a significant age-related change in the frequency of several dendritic cell subsets in the vdLNs, as older mice experienced a decrease in both CD8+ and plasmacytoid DC subsets compared to young controls. Alterations in these DC subsets could limit the generation of a protective immune response. These findings demonstrate that older subjects may not generate fully protective anti-tumor immune responses following mitoxantrone chemotherapy.

